# Methionine and Choline Supply during the Periparturient Period Alter Plasma Amino Acid and One-Carbon Metabolism Profiles to Various Extents: Potential Role in Hepatic Metabolism and Antioxidant Status

**DOI:** 10.3390/nu9010010

**Published:** 2016-12-29

**Authors:** Zheng Zhou, Mario Vailati-Riboni, Daniel N. Luchini, Juan J. Loor

**Affiliations:** 1 Mammalian NutriPhysioGenomics, Department of Animal Sciences and Division of Nutritional Sciences, University of Illinois, Urbana, IL 61801, USA; zzhou32@illinois.edu (Z.Z.); vailati2@illinois.edu (M.V.-R.); 2 Adisseo North America, Alpharetta, GA 30022, USA; daniel.luchini@adisseo.com

**Keywords:** amino acids, lactation, methyl donors, periparturient dairy cow

## Abstract

The objective of this study was to profile plasma amino acids (AA) and derivatives of their metabolism during the periparturient period in response to supplemental rumen-protected methionine (MET) or rumen-protected choline (CHOL). Forty cows were fed from −21 through 30 days around parturition in a 2 × 2 factorial design a diet containing MET or CHOL. MET supply led to greater circulating methionine and proportion of methionine in the essential AA pool, total AA, and total sulfur-containing compounds. Lysine in total AA also was greater in these cows, indicating a better overall AA profile. Sulfur-containing compounds (cystathionine, cystine, homocystine, and taurine) were greater in MET-fed cows, indicating an enriched sulfur-containing compound pool due to enhanced transsulfuration activity. Circulating essential AA and total AA concentrations were greater in cows supplied MET due to greater lysine, arginine, tryptophan, threonine, proline, asparagine, alanine, and citrulline. In contrast, CHOL supply had no effect on essential AA or total AA, and only tryptophan and cystine were greater. Plasma 3-methylhistidine concentration was lower in response to CHOL supply, suggesting less tissue protein mobilization in these cows. Overall, the data revealed that enhanced periparturient supply of MET has positive effects on plasma AA profiles and overall antioxidant status.

## 1. Introduction

Around parturition, the increased demand for nutrients to sustain fetal growth and lactation coupled with depressed dry matter intake (DMI) impose tremendous metabolic stress on dairy cows. Consequently, health problems and compromised production performance likely occur not only due to negative energy balance-induced increases in circulating free fatty acids, but a negative amino acid (AA) balance [1,2,3]. For instance, increased mobilization of tissue protein is often observed due to inadequate availability of AA substrates for gluconeogenesis as well as synthesis of protein in liver and mammary gland [4]. In fact, a moderate net loss of carcass protein was observed even in animals fed to predicted metabolizable protein requirements around parturition [1,5], indicating suboptimal profiles of AA may be the limiting factor for their utilization during this period. In line with this assumption, supplementing rumen-protected limiting AA has achieved various benefits in terms of lactation performance and health status of periparturient dairy cows [6,7,8,9].

Methionine (Met) is an essential sulfur-containing AA associated with various key physiologic events. Previous research has underscored the importance of Met as a limiting AA for milk protein synthesis in many diets [10,11]. Apart from its apparent key role in mammary gland and liver protein synthesis, Met also serves as substrate for sulfur-containing antioxidants, namely glutathione (GSH) and taurine [12]. In addition, as a key component of one-carbon metabolism, hundreds of methylation reactions acquire methyl groups from Met via *S*-adenosyl methionine (SAM) [13]. Furthermore, as a gluconeogenic AA, a portion of Met may be taken up by liver to sustain the abrupt increase in demand for glucose at the onset of lactation. In line with the various biologic processes relying on Met, its deficiency has often been reported in cows around parturition [3]. In fact, circulating Met concentration decreased markedly through parturition and were not restored to prepartum levels until 28 days postpartum [3].

An unfavorable circulating AA profile around parturition likely occurs due to (1) increased production of positive acute-phase proteins (APP) and immune-related proteins induced by oxidative stress and inflammation; (2) enhanced carcass protein mobilization to provide AA for gluconeogenesis; and/or (3) limited uptake of other AA and increased N excretion due to lack of Met. Recent research with periparturient dairy cows has demonstrated benefits to overall health and production performance in response to MET supplementation [7,9,14]. However, knowledge about how plasma AA and downstream products of their metabolism respond to periparturient MET supplementation is lacking.

Although choline (CHOL) is not an AA, it may regulate AA metabolism by altering AA requirements, especially Met, around parturition. For instance, Met can be regenerated when homocysteine receives a methyl group from CHOL through betaine [15,16,17], suggesting that CHOL supplementation can potentially reduce Met requirements around parturition. In addition, accumulation of fat in liver has been speculated to induce inflammation and oxidative stress in ruminant liver, which almost certainly would lead to increased AA requirements for production of positive APP and other immune function-related proteins [17]. As a precursor for hepatic very low-density lipoprotein (VLDL) assembly, CHOL has a crucial role in the export of triacylglycerol to prevent fatty liver by promoting phosphatidylcholine synthesis via the Kennedy pathway instead of sequential methylation using Met derived SAM, which may also spare a portion of Met around parturition.

Few studies have attempted to characterize the profile of circulating AA and their derivatives around parturition in dairy cows [3,18,19,20]. To our knowledge, this is the first study profiling AA and their derivatives in response to MET or CHOL supplementation around parturition. Considering that periparturient MET supplementation resulted in greater feed intake, increased milk yield, and better overall cow health while CHOL cows did not achieve similar benefits [9] (although others have reported increased milk production with CHOL [21,22]), our hypothesis was that MET and CHOL supplementation results in different alterations in AA metabolism-associated events which ultimately contribute to their distinct roles in the overall health and production efficiency of the animal.

## 2. Materials and Methods

### 2.1. Experimental Design and Treatments

All procedures for this study (protocol no. 13023) were approved by the Institutional Animal Care and Use Committee (IACUC) of the University of Illinois. Details of the experimental design have been described previously [9,23]. Briefly, the experiment was conducted as a randomized, complete, unbalanced, block design with 2 × 2 factorial arrangement of MET (Smartamine M, Adisseo NA, Alpharetta, GA, USA) and CHOL (ReaShure, Balchem Inc., New Hampton, NY, USA) level (with or without). Cows within each block were balanced for parity, previous lactation milk yield, and BCS before the close-up diet groups were assigned. A total of 81 cows were used. Treatments were control (CON, *n* = 20), with no MET or CHOL supplementation; Smartamine (SMA, *n* = 21), CON plus MET at a rate of 0.08% of DM; Reashure (REA, *n* = 20), CON + CHOL at 60 g/days; or Smartamine and Reashure (MIX, *n* = 20), CON + MET + CHOL. Dosage of MET was based on Osorio et al. [6], whereas CHOL was supplemented following the manufacturer’s recommendations. Met as a % of metabolizable protein (MP) and the Lys:Met ratio for close-up diets were estimated to be 1.9% and 3.6:1 Lys:Met for CON, 2.4% and 2.8:1 Lys:Met for SMA, 1.9% and 3.6:1 Lys:Met for REA, and 2.4% and 2.8:1 Lys:Met for MIX. Met as a % of MP and the Lys:Met ratio for lactation diets were estimated to be 1.8% and 3.5:1 Lys:Met for CON, 2.3% and 2.7:1 Lys:Met for SMA, 1.8% and 3.5:1 Lys:Met for REA, and 2.3% and 2.7:1 Lys:Met for MIX. Per IACUC conclusions, a subset of 40 multiparous cows (10 cows/treatment) was deemed sufficient to achieve statistical power. Thus, these cows were selected randomly and used for this portion of the study. All cows received the same far-off diet from −50 to −22 days before expected parturition, the close-up diet from −21 days to expected parturition, and the lactation diet from parturition through 30 days in milk (DIM). The MET and CHOL supplements were both top-dressed from −21 ± 2 to 30 DIM once daily at the AM feeding using approximately 50 g of ground corn as carrier for all treatments. On average, cows received MET and/or CHOL supplementation for 23.1 ± 1.0 days prepartum. Supplementation of SMA (0.08% DM of TMR offered) was calculated daily for each cow. Smartamine M was supplied as small beads containing a minimum of 75% DL-Methionine (DL-Met), physically protected by a pH sensitive coating, which is considered to have a Met bioavailability of 80% [24]; therefore, per 10 g of SMA, the cows received 6 g of metabolizable Met. The REA supplement is reported to contain 28.8% choline chloride and is protected by microencapsulation. In terms of bioavailability, work from a graduate thesis using (methyl,^2^H3)-CHOL and (methyl,^2^H3)-Met indicated, based on differences in Met methyl flux rates, that the product has CHOL bioavailability of 72% [25]; therefore, per 60 g of REA, cows in our study would have received 12.4 g of metabolizable choline chloride. In contrast, a recent in vivo study evaluating bioavailability of REA detected low portal flux of free choline (13%) relative to abomasal delivery of choline [26], thus, indicating that only 2.3 g of free choline would have been available post-ruminally. Although assessing bioavailability of SMA and REA was beyond the scope of our study, it is important to note that the approaches for estimating “bioavailability” of CHOL in these studies was different. For instance, the work of de Veth et al. [26] did not consider the portion of CHOL partly oxidized to betaine, partly phosphorylated, and partly incorporated into lyso- and phosphatidylcholine within the enterocyte and prior to entering portal circulation. Thus, despite the low concentration of free choline any betaine generated during metabolism within enterocytes could have contributed methyl groups for remethylation of homocysteine to Met in the liver. Clearly, further studies would have to be conducted to define better CHOL needs of periparturient cows and the efficacy of available CHOL products in delivering metabolizable CHOL and its derivatives. To our knowledge, neither SMA nor REA have specific characteristics that may affect palatability of diets.

### 2.2. Animal Management

Dry cows were housed in a ventilated enclosed barn during the dry period and fed individually once daily at 06:30 am using an individual gate system (American Calan Inc., Northwood, NH, USA). After parturition, cows were housed in a tie-stall barn and were fed a common lactation diet once daily. Feed offered was adjusted daily to achieve ~10% refusals.

### 2.3. Blood Sample Collection and Analyses of Plasma AA and Their Derivatives

Blood was sampled from the coccygeal vein at −30 and −10 days relative to expected parturition date and at 4, 14 and 28 days relative to actual parturition date before the AM feeding. On average, prepartum samples were harvested at −30.8 ± 1.4 days and −10.9 ± 1.3 days relative to actual calving date. Samples were collected into evacuated tubes (BD Vacutainer; BD and Co., Franklin Lakes, NJ, USA) containing lithium heparin for isolation of plasma.

Plasma was used to analyze the concentrations of free arginine (Arg), histidine (His), isoleucine (Ile), leucine (Leu), lysine (Lys), methionine (Met), phenylalanine (Phe), threonine (Thr), tryptophan (Trp), valine (Val), asparagine (Asn), aspartate (Asp), alanine (Ala), glutamate (Glu), glutamine (Gln), glycine (Gly), proline (Pro), serine (Ser), tyrosine (Tyr), citrulline (Cit), carnosine, ornithine (Orn), sarcosine (Sar), cystathionine, cystine, homocystine, taurine, α-aminoadipic acid, α-aminobutyric acid, β-alanine, γ-aminobutyric acid (GABA), hydroxylysine, hydroxyproline, phosphoserine, 1-methyl histidine, and 3-methyl histidine at the University of Missouri Agriculture Experiment Station Chemical Laboratories (Columbia, MO, USA) using high performance liquid chromatography [27,28]. Plasma total GSH was measured using a commercial kit (Cat. No. NWH-GSH01; Northwest Life Science Specialties LLC, Vancouver, WA, USA). The essential amino acid (EAA) pool included Arg + His + Ile + Leu + Lys + Met + Phe + Thr + Trp + Val; the NEAA pool included Asn + Asp + Ala + Gln + Glu + Gly + Pro + Ser + Tyr; total AA was the sum of EAA and non-essential amino acid (NEAA); the total sulfur-containing compounds (TSC) included Met + cystine + cystathionine + homocysteine + taurine + GSH.

### 2.4. Liver Sample Collection and Quantitative RT-PCR (qPCR)

Liver was sampled via puncture biopsy [29] from cows under local anesthesia at approximately 08:00 am on days −10, 7, 20, and 30 days relative to parturition. Liver was frozen immediately in liquid nitrogen and stored until analysis. The qPCR was performed in liver samples as described previously [7].

### 2.5. Statistical Analysis

Data were analyzed using PROC MIXED of SAS (SAS Institute Inc., Cary, NC, USA) according to the following model:

$$Y_{ijklm}=\mu +b_{i}+M_{j}+C_{k}+MC_{jk}+T_{l}+TM_{jl}+TC_{kl}+TMC_{jkl}+A_{m:ijk}+\varepsilon_{ijklm}$$

where $Y_{ijklm}$ is the dependent, continuous variable; μ is the overall mean; $b_{i}$ is the random effect of the ith block; $M_{j}$ is the fixed effect of MET (j = with or without);$\;C_{k}$ is the fixed effect of CHOL (k = with or without); $T_{l}$ is the fixed effect of time (day or week) of the experiment; $A_{m}$ is the random effect of the *m*th animal (cow); $\varepsilon_{ijklm}\;$is the residual error. The covariate of parity (2nd vs. 3rd lactation and above) and concentrations obtained at −30 days for various AA and derivatives were maintained in the model for all variables when significant (*p* < 0.05). Plasma AA and derivatives and hepatic gene expression were analyzed at various time points that were not equally spaced. Therefore, the first order ante-dependence covariance structure 1 (ANTE(1)) was used for repeated measures. Variables were assessed for normality of distribution using the Shapiro-Wilk test. When the normality assumption was rejected, data were log-transformed before statistical analysis. Back transformed data are reported in tables and figures for ease of interpretation. Data reported are least square means for each time point and least square means separation between treatments and time points were performed using the PDIFF statement. Statistical differences were declared significant at *p* < 0.05 and tendencies at *p* < 0.10.

## 3. Results

### 3.1. Essential AA

**Figure 1:**
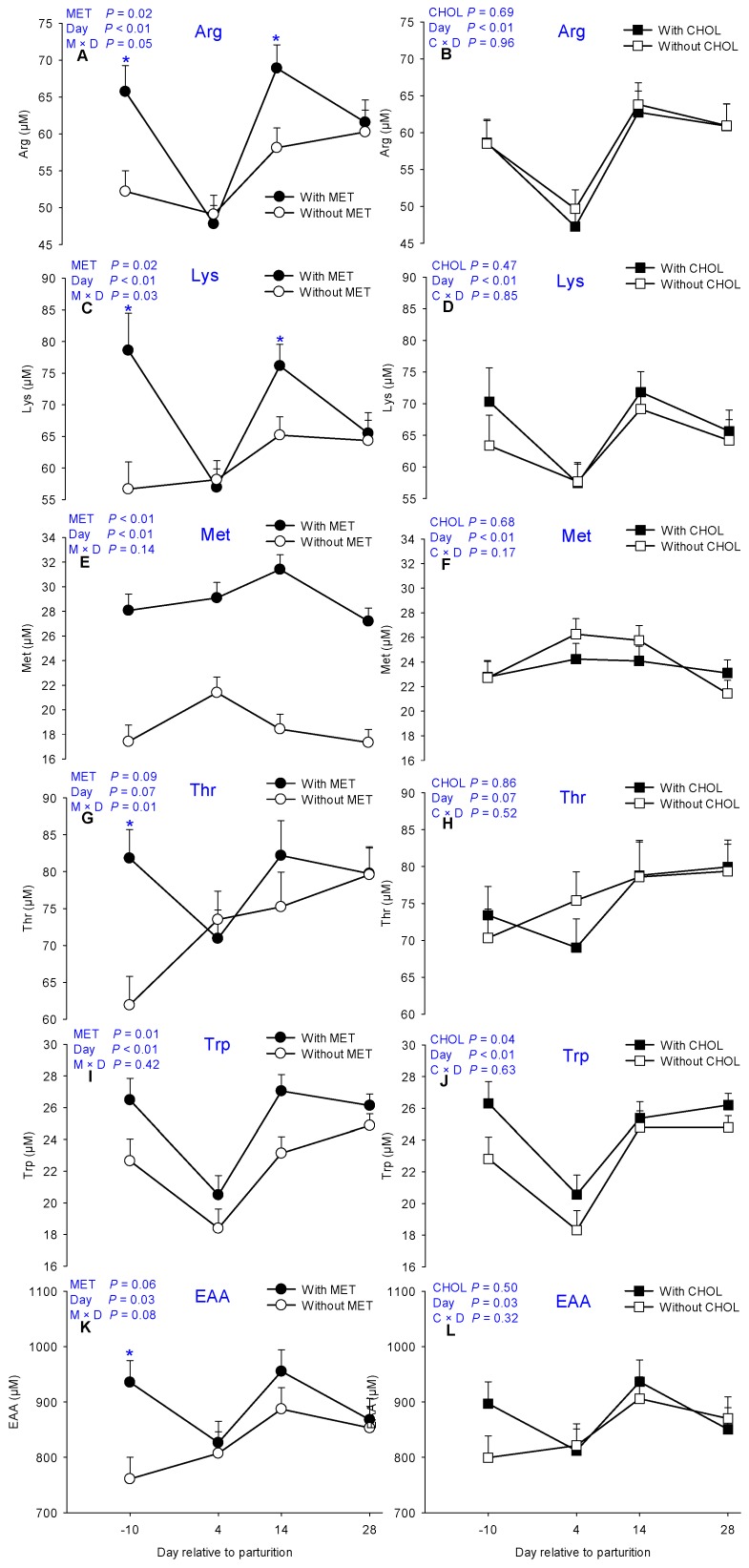
Effects of supplementing multiparous Holstein cows during the periparturient period (−21 through 30 days around parturition) with rumen-protected methionine (MET; Smartamine M, Adisseo NA, Alpharetta, GA, USA) or rumen-protected choline (CHOL; ReaShure, Balchem Inc., New Hampton, NY, USA) on circulating concentrations of arginine, lysing, methionine, threonine, tryptophan, and total essential AA (EAA). Values are means, with standard errors represented by vertical bars.

**Figure 2:**
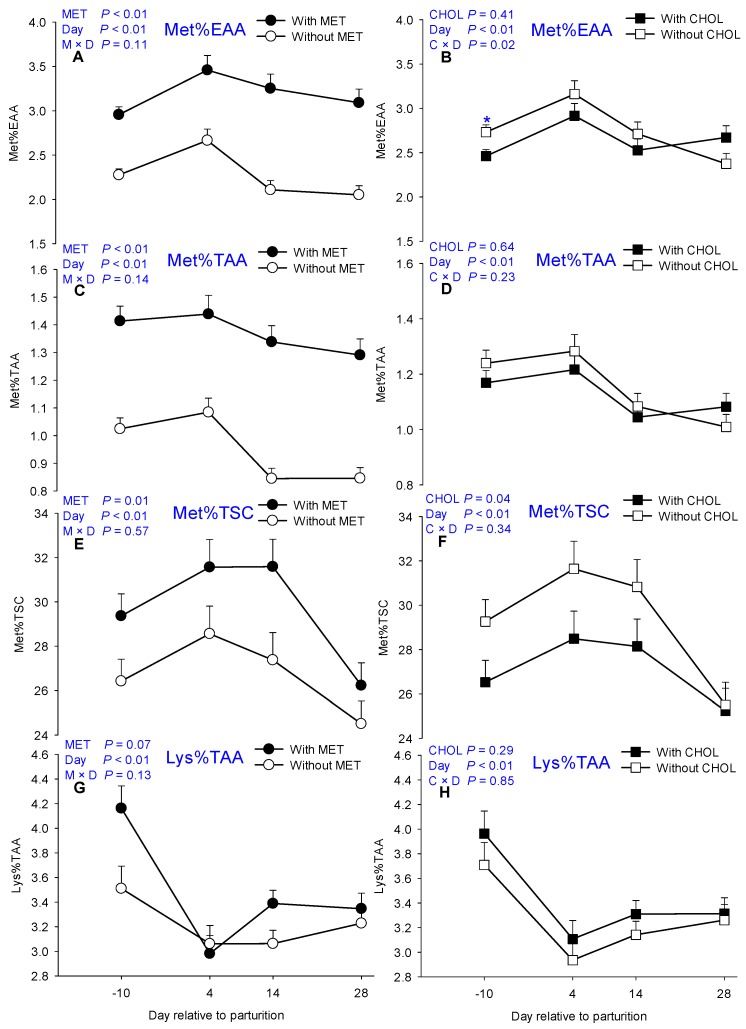
Effects of supplementing multiparous Holstein cows during the periparturient period (−21 through 30 days around parturition) with rumen-protected methionine (MET; Smartamine M, Adisseo NA) or rumen-protected choline (CHOL; ReaShure, Balchem Inc.) on proportions of circulating Met in essential amino acids (Met%EAA), total amino acids (Met%TAA), total sulfur-containing compounds (Met%TSC), and proportion of Lys in TAA (Lys%TAA). Values are means, with standard errors represented by vertical bars.

Main effects of MET, CHOL, and their interactions with time for essential AA are presented in Table 1. Means for MET × CHOL are presented in Table S1. Overall, plasma concentrations of Arg, His, Lys, and Trp decreased soon after parturition (4 days). In contrast, circulating Met concentration increased at 4 days (Figure 1). As expected, MET-supplemented cows had greater plasma Met concentration at all time points (*p* < 0.01, Figure 1E) compared with cows without MET. In fact, the proportion of Met in EAA (*p* < 0.01, Figure 2A) and total AA pool (TAA) (*p* < 0.01, Figure 2C) also was greater in response to MET supplementation. In contrast, plasma Met (*p* > 0.10, Figure 1F) and Met%EAA (*p* > 0.10, Figure 2B) levels were not different in response to CHOL supplementation. A main effect of MET (*p* = 0.02) also was detected for Lys, the second most-limiting AA for milk production, mainly due to greater plasma concentrations at −10 days and 14 days (M × D *p* < 0.05, Figure 1C). Similarly, plasma Arg concentration also was greater (*p* = 0.02) in MET cows owing to greater concentrations at −10 days and 14 days (M × D *p* < 0.05, Figure 1A), but not at 4 days postpartum. Although greater Thr was detected in MET cows at −10 days (M × D *p* < 0.05, Figure 1G), Thr concentrations were not greater at other time points measured postpartum in MET-supplemented cows, hence, overall only a tendency (*p* = 0.09) was detected in these cows. In addition to greater (*p* < 0.01) Trp in MET cows, a main effect of CHOL also was detected for Trp (*p* = 0.04, Figure 1I,J). Despite the lack of main effects of MET for plasma His, Phe and total branched-chain amino acids (BCAA = sum of Val, Leu, and Ile) concentrations, the EAA tended to be greater (*p* = 0.06) in response to MET mainly owing to greater EAA concentration prepartum (M × D *p* < 0.05, Figure 1K).

### 3.2. Non-Essential AA

**Figure 3:**
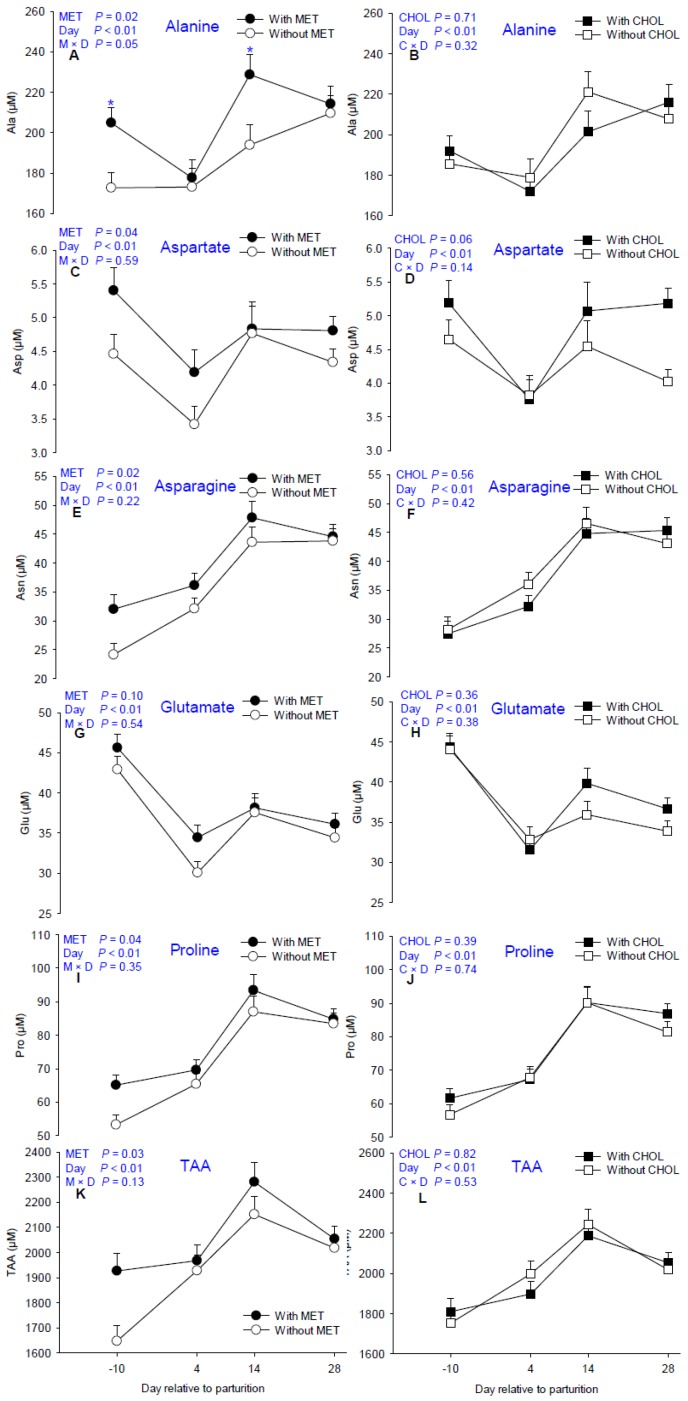
Effects of supplementing multiparous Holstein cows during the periparturient period (−21 through 30 days around parturition) with rumen-protected methionine (MET; Smartamine M, Adisseo NA) on circulating concentrations of free alanine, aspartate, asparagine, glutamate, proline, and total AA (TAA). Values are means, with standard errors represented by vertical bars.

Main effects of MET, CHOL, and their interactions with time for non-essential proteinogenic AA are presented in Table 1. Means for MET × CHOL are presented in Table S1. Similar to EAA, plasma concentrations of Asp, Glu, Gln, and Tyr decreased soon after parturition (Figure 3). In contrast, plasma concentrations of Asn, Gly, Pro, and Ser increased at 4 days compared with −10 days. Although total NEAA did not change (*p* > 0.10) in response to MET or CHOL, TAA in MET-supplemented cows was greater (*p* = 0.03, Figure 3K) as a result of an overall tendency for greater EAA together with greater Asn, Asp, Ala, and Pro (*p* < 0.05, Figure 3A,C,E,I) as well as a tendency for greater Glu (*p* = 0.10, Figure 3G).

It is noteworthy that similarly to Arg and Lys (M × D *p* < 0.05, Figure 1A,C), plasma Ala concentrations were already greater (M × D *p* < 0.05, Figure 3A) at −10 days prepartum in MET cows. After parturition, plasma Arg, Lys, and Ala were not different regardless of MET supplementation at 4 days, but were again greater at 14 days (M × D *p* < 0.05, Figure 1A,C and Figure 3A) in MET cows. Unlike Met, only a tendency for greater (*p* = 0.06, Figure 3D) Asp was detected in response to CHOL.

### 3.3. Sulfur-Containing Compounds

**Figure 4:**
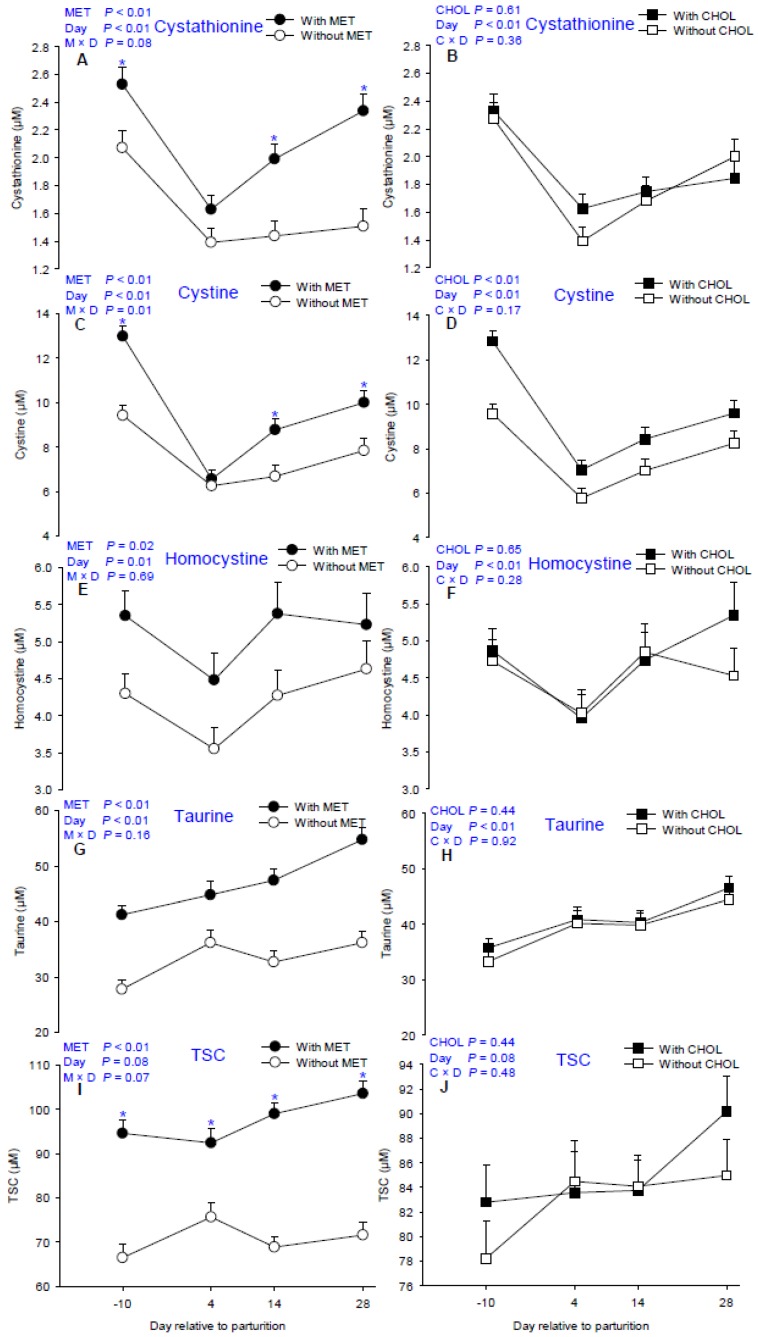
Effects of supplementing multiparous Holstein cows during the periparturient period (−21 through 30 days around parturition) with rumen-protected methionine (MET; Smartamine M, Adisseo NA) on circulating concentrations of cystathionine, cystine, homocysteine, taurine, and total sulfur-containing compounds (TSC). Values are means, with standard errors represented by vertical bars.

Main effects of MET, CHOL, and their interactions with time for sulfur-containing compounds are presented in Table 2. Means for MET × CHOL are presented in Table S2. As expected, MET supplementation increased plasma concentrations of cystathionine, cystine, homocysteine, and taurine (*p* < 0.05, Figure 4A,C,E,G), which account for the greater overall plasma TSC level at all time points evaluated (M × D *p* < 0.05, Figure 4I). Similar to Ala, Arg, and Lys, greater (*p* < 0.01) cystine and cystathionine concentrations were also detected in response to MET due to greater plasma level at −10, 14, and 28 days (M × D *p* < 0.05, Figure 4A,C). In addition to increased circulating sulfur-containing compounds, MET also increased the proportion of Met in TSC (*p* = 0.01, Figure 2E). In contrast, although plasma cystine concentration was greater (*p* < 0.01, Figure 4D) in response to CHOL supplementation, a decreased proportion of Met in TSC was detected in CHOL cows around parturition (*p* = 0.03, Figure 2F).

### 3.4. Non-Proteinogenic AA and Derivatives

**Figure 5:**
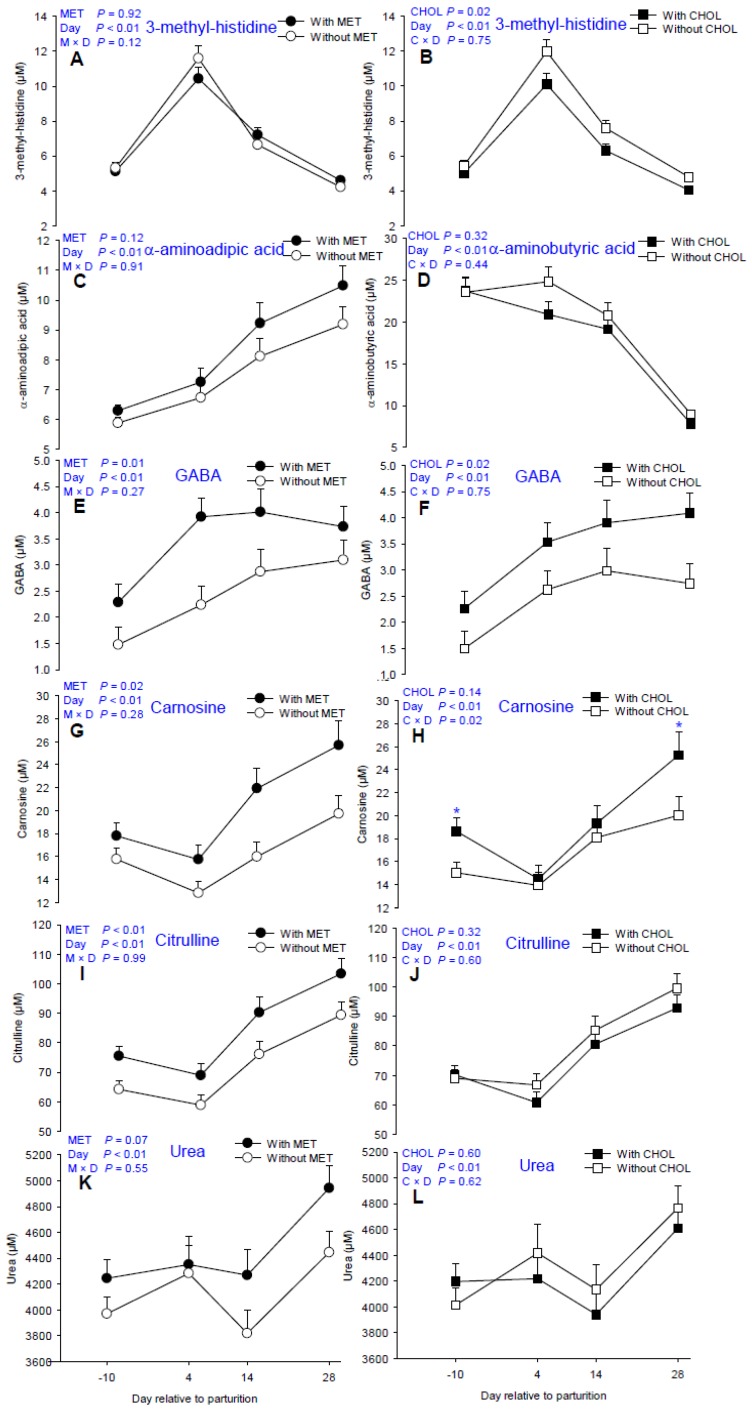
Effects of supplementing multiparous Holstein cows during the periparturient period (−21 through 30 days around parturition) with rumen-protected methionine (MET; Smartamine M, Adisseo NA) or rumen-protected choline (CHOL; ReaShure, Balchem Inc.) on proportions of circulating 1-methyl histidine, 3-methyl histidine, γ-amino butyric acid, carnosine, citrulline, and urea. Values are means, with standard errors represented by vertical bars.

Main effects of MET, CHOL, and their interactions with time for non-proteinogenic AA and derivatives are presented in Table 2. Means for MET × CHOL are presented in Table S2. Although no main effect of MET or CHOL was detected for ornithine, greater Cit (*p* < 0.01, Figure 5I) and a tendency (*p* = 0.07, Figure 5K) for greater urea were observed in MET-supplemented cows in addition to greater Arg. Besides taurine, plasma concentrations of carnosine, another antioxidant, was also greater (*p* = 0.02, Figure 5G) in MET cows. Although no main effect of CHOL was detected for carnosine, greater (C × D *p* < 0.05, Figure 5H) concentrations were detected at −10 days and 28 days. γ-aminobutyric acid was greater both in MET- and CHOL-supplemented cows (*p* < 0.05, Figure 5E,F). The indicator of protein mobilization, 3-methylhistidine, was lower in CHOL cows (*p* = 0.02, Figure 5D). Similarly, plasma 1-methylhistidine concentration also was lower in these cows (*p* = 0.04).

### 3.5. Pyruvate Carboxylase and Phosphoenolpyruvate Carboxykinase 1 Expression

Main effects of MET, CHOL, and their interactions with time for expression of *pyruvate carboxylase* (*PC*) and *phosphoenolpyruvate carboxykinase 1* (*PCK1*) are presented in Table 3. Means for MET × CHOL are presented in Table S3. Main effects of MET and CHOL or interactions were not detected (*p* > 0.05) for the mRNA expression of *PC* and *PCK1.*

## 4. Discussion

A detailed discussion of production performance and immunometabolic status of the cows has been published elsewhere [8,9,23].

### 4.1. Enhancing the Supply of MET Improved Plasma AA Profiles

MET and Lys in the MP are the most-limiting AA in a wide-range of diets for dairy cows due to their low concentrations in feed protein relative to their “apparent required amounts in digestible protein” [30]. During the periparturient period, the decreased feed intake coupled with increased AA requirements to sustain fetal growth and lactation lead to greater tissue protein mobilization evidenced in the present study by the highest plasma 3-methyl histidine at 4 days regardless of treatments. Although AA released from tissue mobilization can partly mitigate the demand for AA, cow body tissue protein is relatively low in Met and Lys [31]. Consequently, this physiological stage is characterized by an overall decrease in circulating AA, especially the most-limiting AA (e.g., Met and Lys). Apart from limiting milk production, according to von Liebig’s hypothesis commonly described with the analogy of a water barrel with broken staves, inadequate Met availability also could potentially limit the utilization of other circulating AA [32]. Strategies to increase circulating Met are, thus, expected to improve overall circulating AA profiles and utilization during the periparturient period.

Apart from greater plasma Met, the fact that various proteinogenic circulating EAA (Arg, Lys, Met, Thr, and Trp), NEAA (Ala, Asn, Asp, Glu, Gln, and Pro) and non-proteinogenic AA (Cit) were greater in response to rumen-protected MET supplementation is an indication of a better circulating AA profile during the periparturient period. However, it cannot be ignored that MET cows had greater DMI both prepartum and postpartum [9,23]. Therefore, the DMI increase in MET-supplemented cows might have contributed to the observed increase in circulating EAA and TAA. It is noteworthy that despite concomitant increases in circulating Met, EAA and TAA, the proportion of Met in EAA and TAA (Met%EAA and Met%TAA) as well as the proportion of Lys in TAA were increased in response to MET supplementation, indicating a better AA profile at least in regards to limiting AA, i.e., Met and Lys.

Considering the overall greater milk yield, milk protein %, and milk protein yield [9,23], it is reasonable to speculate that MET supplementation contributes to better lactation performance both by increasing intake and proportion of Met and Lys in the circulating AA pool. Although CHOL can, in theory, promote in vivo synthesis of Met or indirectly spare Met by reducing the use of Met for CHOL synthesis, the lack of change in circulating AA in the present study and the fact that flux from CHOL to MET was not increased in response to CHOL supplementation in lactating goats [33] and transition dairy cows [34] do not support such hypothesis.

### 4.2. Utilization of Circulating AA Close to Parturition

Around parturition, the demand for AA for glucose and protein synthesis increases abruptly and results in greatly increased AA uptake from the circulation [35]. The few published reports have revealed that circulating AA concentrations in dairy cows generally reach a nadir close to parturition mainly as a result of increased demand for milk protein synthesis and gluconeogenesis coupled with insufficient dietary intake [3,18,20,36]. In agreement with these reports, regardless of treatment, we detected the lowest circulating concentrations of most AA at 4 days relative to parturition.

Although circulating concentrations of multiple AA (Ala, Arg, Lys) and derivatives (cystine, cystathionine) in MET-supplemented cows were already greater at −10 days compared with cows without MET supplementation and regained greater levels at 14 days postpartum, the circulating concentrations of all AA and derivatives at 4 days were similar regardless of MET supplementation. Whether the lack of difference in circulating concentrations of these AA at 4 days in MET cows was due to inadequate supply in the circulation (e.g., AA from intake and tissue mobilization) [37] or enhanced utilization (e.g., greater liver and mammary uptake) remains unknown. However, the fact that average DMI at day 4 remained greater (+3.33 kg/days more) in MET-supplemented cows [9,23], together with similar 3-methylhistidine in cows with or without MET supplementation, seems to support an overall greater, rather than lower, AA supply at 4 days in response to MET supplementation. In line with the increase in AA supply, MET-supplemented cows regained greater concentrations of these AA at 14 days.

In terms of AA utilization, a previous study detected the greatest liver uptake of EAA at 4 days postpartum in dairy cows [35]. Similarly, hepatic uptake of total NEAA, especially Ala, also was substantially greater in the periparturient period, underscoring that this AA acts as a precursor for glucose synthesis [35,38]. The greater (+4.10 kg/day more) milk yield in MET-supplemented cows at 4 days [9,23] could have caused an increase in uptake of these AA by mammary gland soon after parturition, hence, resulting in lower plasma AA concentration relative to −10 days. Presumably, greater mammary availability of AA accounted for the greater milk protein % [9,23].

It is also noteworthy that a reduction in net flux of carbon from volatile fatty acids across the liver and an increase in glucose release was detected early postpartum in a previous report [35], indicating that the greatest flux through citric acid cycle occurs soon after parturition. Considering the severe negative energy balance and depressed DMI soon after parturition, the increased demand for energy and glucose synthesis by the liver likely accounted for the increase in citric acid flux. Whether periparturient MET supplementation in the present study enhanced gluconeogenesis by promoting AA flux through the citric acid cycle is unknown, but does not seem to be related to regulation at the transcriptional level as evidenced by unchanged mRNA abundance of the key gluconeogenic genes *PC* and *PCK1.*

### 4.3. Sulfur-Containing Compound Pool and Metabolism

Met and cysteine are the two sulfur-containing AA that are incorporated into proteins. Apart from their well-known role in contributing sulfur bonds during protein synthesis, Met and cysteine are precursors for downstream functional compounds (homocysteine, cystathionine, and taurine) and, thus, are considered the principal components in vivo of the sulfur-containing compound pool [12]. The fact that circulating concentrations of all sulfur-containing compounds measured (except GSH) were greater in MET-supplemented cows during the periparturient period indicate an enriched sulfur-containing compound pool.

It is noteworthy that other than Met, all other plasma sulfur-containing compounds measured are components of the transsulfuration pathway. Considering plasma homocysteine concentrations are highly-dependent on intracellular homocysteine metabolism in liver [39], and cystathionine is a sensitive marker of changes in flux through the transsulfuration pathway [40], the greater homocysteine and cystathionine concentrations indicate increased hepatic flux through this pathway in response to MET supplementation. In addition to the involvement of sulfur-containing compounds, the fact that circulating concentrations of α-aminobutyric acid also were greater in MET-supplemented cows indicates increased flux through the transsulfuration pathway [41]. Considering the key role of Met as the most-limiting AA for milk protein synthesis, the fact that it is the major precursor for sulfur-containing compounds in the transsulfuration pathway indicates that such increase may result in depletion of Met which could potentially give rise to unfavorable lactation performance. However, despite the increased flux through the transsulfuration pathway, the sustained greater circulating concentration of Met and proportion of Met in TSC during the periparturient period indicates that sufficient Met was available in MET-supplemented cows even at 4 days. In contrast, although greater circulating cystine was detected in response to CHOL supplementation, the proportion of Met in TSC was decreased, indicating enhanced flux through the transsulfuration pathway at the expense of Met. Whether the decrease of plasma Met proportion in TSC in the CHOL-supplemented cows contributed to the lack of benefit in performance is unknown; however, it does not seem to support the hypothesis that CHOL can promote Met synthesis in vivo in periparturient dairy cows.

Around parturition, the increased demand for nutrients and energy leads to an increase in the production reactive oxygen metabolites (ROM), the accumulation of which could deplete antioxidants and give rise to oxidative stress that may cause substantial tissue damage and render cows more susceptible to various health disorders [42,43]. Because of their marked ability to scavenge ROM and free radicals, among the sulfur-containing compounds measured, taurine and GSH are considered potent intracellular antioxidants [44]. Hence, in non-ruminants, hepatic concentrations of GSH and taurine have been widely-used as oxidative stress biomarkers [45,46,47]. Because the liver is the main site of taurine synthesis and releases it into plasma [48], its concentration in the circulation reflects hepatic synthesis [49]. Therefore, the greater overall circulating taurine in response to MET supplementation indicates greater hepatic and extra-hepatic taurine availability and, hence, potentially less oxidative stress in these cows. However, although previous results from our group revealed greater total and reduced hepatic GSH in MET-supplemented cows [14,50], plasma GSH was barely detectable and did not respond to MET. Considering that concentration of GSH in whole blood was 200-fold higher than in plasma due to high concentration in erythrocytes, small amounts of hemolysis may lead to great variations in plasma GSH concentration [51]. Therefore, plasma GSH may not be a reliable oxidative stress biomarker for periparturient dairy cows.

### 4.4. AA Derivatives

AA derivatives have unique metabolic properties and, thus, many have been adopted as biomarkers for metabolic status. For instance, because 3-methylhistidine is released from the catabolism of actin and myosin in skeletal muscle and is not further metabolized in the body, it has been regarded as a reliable marker for tissue protein mobilization [52]. Assuming that renal blood flow was not altered by CHOL supplementation, the overall lower plasma 3-methylhistidine in response to CHOL supplementation during the periparturient period indicates a lower degree of muscle catabolism. Considering that CHOL supplementation did not result in greater milk yield, the lower degree of tissue mobilization could denote better production efficiency. However, the fact that CHOL supplementation did not alter energy corrected milk (ECM):DMI and fat corrected milk (FCM):DMI does not support such hypothesis. Although lower circulating 1-methylhistidine concentration also was observed in response to CHOL, the postpartum pattern of a gradual increase with time indicates that it is not a suitable indicator of muscle catabolism considering that it was at its greatest right after parturition.

The antioxidant activity of carnosine in non-ruminants has been demonstrated both in terms of reducing oxidative damage and improving the enzymatic and non-enzymatic activity of other antioxidants [53]. The fact that supplementation of carnosine in non-ruminants was able to rescue the prooxidant-antioxidant balance by restoring depleted levels of blood GSH and activities of antioxidant enzymes [54] indicates that circulating carnosine concentrations could decrease in events of oxidative stress. Although the contribution of carnosine to restoring prooxidant–antioxidant balance under physiologic (without carnosine supplementation) conditions remains unknown, the overall greater circulating carnosine together with greater circulating taurine as well as hepatic GSH reported previously [8] indicate a less pronounced oxidative stress status in response to periparturient MET supplementation.

As a neurotransmitter distributed in both neural and non-neural tissue, GABA has various physiologic functions including feed intake regulation [55]. In rats, GABA-B agonist administration increased intake by attenuating satiety signals [56]. In lactating dairy cows, increased DMI in response to rumen-protected GABA supplementation during mid-lactation has been reported [55,57]. The greater circulating GABA in response to periparturient MET supplementation along with the greater DMI [9,23] seem to suggest a role of GABA in mediating DMI regulation by MET. However, considering that CHOL supplementation failed to increase periparturient DMI, yet resulted in a similar increase in circulating GABA, seems to argue against a regulatory role of this molecule in the control of intake at least around parturition.

## 5. Conclusions

The greater Ala, Arg, and Lys together with cystine and cystathionine concentrations at −10 days and 14 days, but not at 4 days, in cows fed MET indicated greater utilization of these AA and derivatives at the time of most-severe negative AA balance. The enriched circulating sulfur-containing compound pool together with greater α-aminobutyric acid revealed enhanced transsulfuration pathway activity in response to MET supplementation. Despite the greater circulating cystine in CHOL cows, the lower proportion of Met in TSC indicates enhanced flux through the transsulfuration pathway at the expense of Met. Whether the decrease in the proportion of Met was due to insufficient choline supplementation remains unknown. As a precursor for Met synthesis in vivo, it is possible that increasing periparturient choline supplementation could mitigate a decrease in Met. Although plasma GSH did not differ in response to MET due to barely detectable concentrations in plasma, the greater circulating taurine and carnosine indicate less oxidative stress in MET-supplemented cows. In conclusion, the overall better health and production performance reported previously in MET cows was due, at least in part, to a better plasma AA profile and overall lower oxidative stress status. Hence, the lack of change in AA profiles could be one of the reasons preventing CHOL-fed cows from achieving comparable performance and health benefits during the periparturient period.

## Figures and Tables

**Table 1:** Plasma proteinogenic AA concentrations during the periparturient period in cows supplemented with or without rumen-protected MET and CHOL.

	MET		CHOL			Day				*p*-Value ^2^				
AA (µM)	Without	With	Without	With	SEM ^1^	−10	4	14	28	MET ^3^	CHOL ^4^	Time	M × T ^5^	C × T ^6^
Essential AA														
Arginine	54.75	60.43	57.99	57.05	1.75	58.58 ^ab^	48.47 ^c^	63.29 ^a^	60.92 ^ab^	0.02	0.69	<0.01	0.05	0.96
Histidine	52.38	54.55	54.04	52.87	1.18	57.28 ^a^	53.53 ^b^	52.30 ^b^	50.92 ^b^	0.19	0.49	<0.01	0.13	0.17
Isoleucine	95.58	103.61	100.64	98.40	4.42	98.18	95.54	106.87	97.83	0.19	0.72	0.18	0.07	0.36
Leucine	153.31	161.79	152.70	162.43	6.69	151.35 ^b^	153.51 ^b^	173.18 ^a^	152.92 ^b^	0.35	0.31	0.03	0.27	0.31
Lysine	60.99	68.74	63.47	66.06	2.45	66.75 ^ab^	57.56 ^c^	70.47 ^a^	64.91 ^b^	0.02	0.47	<0.01	0.03	0.85
Methionine	18.65	28.95	24.04	23.55	0.83	22.75 ^c^	25.25 ^a^	24.92 ^ab^	22.27 ^c^	<0.01	0.68	<0.01	0.14	0.17
Phenylalanine	46.64	47.09	45.88	47.86	1.02	45.00 ^b^	47.64 ^b^	51.16 ^a^	43.97 ^b^	0.75	0.17	<0.01	0.26	0.92
Threonine	72.55	78.67	75.93	75.30	2.50	71.87	72.22	78.70	79.66	0.09	0.86	0.07	0.01	0.52
Tryptophan	22.26	25.04	22.68	24.61	0.64	24.56 ^ab^	19.44 ^c^	25.08 ^a^	25.50 ^a^	<0.01	0.04	<0.01	0.42	0.63
Valine	226.77	244.54	229.16	241.99	9.32	230.11	223.02	249.41	240.25	0.17	0.33	0.08	0.26	0.31
BCAA ^7^	476.69	510.97	483.71	503.55	19.87	480.67	473.17	530.28	491.93	0.21	0.48	0.08	0.19	0.27
EAA	827.12	896.20	849.22	874.1	25.54	848.26 ^bc^	816.78 ^bc^	921.2 ^a^	860.39 ^ab^	0.06	0.50	0.03	0.08	0.32
Met%EAA	2.26	3.18	2.73	2.64	0.09	2.59 ^b^	3.04 ^a^	2.62 ^b^	2.52 ^b^	<0.01	0.41	<0.01	0.11	0.23
Non-essential AA														
Alanine	187.39	206.37	198.37	195.39	5.55	188.83 ^b^	175.45 ^c^	211.30 ^a^	211.95 ^a^	0.02	0.71	<0.01	0.05	0.32
Asparagine	34.90	39.63	37.78	36.61	1.48	27.82 ^c^	34.08 ^b^	45.67 ^a^	44.19 ^a^	0.02	0.56	<0.01	0.22	0.42
Aspartate	4.21	4.79	4.24	4.76	0.21	4.91 ^a^	3.78 ^b^	4.80 ^a^	4.57 ^a^	0.04	0.07	<0.01	0.59	0.14
Glutamate	35.93	38.34	36.44	37.81	1.04	44.24 ^a^	32.17 ^d^	37.84 ^b^	35.24 ^c^	0.10	0.36	<0.01	0.54	0.38
Glutamine	246.22	259.53	257.92	247.82	6.18	278.17 ^a^	252.84 ^b^	242.52 ^b^	237.96 ^b^	0.15	0.25	<0.01	0.21	0.29
Glycine *	426.83	406.23	427.39	405.67	15.10	236.99 ^c^	456.97 ^b^	536.19 ^a^	435.98 ^b^	0.35	0.31	<0.01	0.92	0.22
Proline	72.30	78.24	74.03	76.50	2.02	59.19 ^c^	67.52 ^b^	90.21 ^a^	84.15 ^a^	0.04	0.39	<0.01	0.35	0.74
Serine	86.62	88.92	88.67	86.87	2.28	73.88 ^c^	90.85 ^b^	100.78 ^a^	85.57 ^b^	0.48	0.58	<0.01	0.16	0.49
Tyrosine	40.82	43.39	40.78	43.43	1.53	42.03 ^a^	36.26 ^b^	46.32 ^a^	43.80 ^a^	0.24	0.24	<0.01	0.08	0.94
NEAA ^8^	1114.59	1151.58	1153.58	1112.59	24.79	946.13 ^c^	1136.83 ^b^	1301.27 ^a^	1177.00 ^b^	0.29	0.24	<0.01	0.36	0.23
TAA ^9^	1926.95	2052.97	1995.87	1982.22	41.32	1781.41 ^d^	1947.89 ^bc^	2215.47 ^a^	2035.97 ^b^	0.03	0.82	<0.01	0.10	0.53
Met%TAA	0.94	1.37	1.15	1.13	0.04	1.20 ^a^	1.25 ^a^	1.06 ^b^	1.05 ^b^	<0.01	0.64	<0.01	0.14	0.23
Lys%TAA	3.22	3.47	3.26	3.42	0.10	3.84 ^a^	3.02 ^c^	3.23 ^bc^	3.29 ^b^	0.08	0.28	<0.01	0.13	0.85

* Significant (*p* < 0.05) parity effect observed. Labeled means in a row without a common superscript letter differ (*p* < 0.05). ^1^ Greatest SEM; ^2^ MET × CHOL interaction was included in the supplemental file; ^3^ Overall effect of MET supplementation; ^4^ Overall effect of CHOL supplementation; ^5^ Interaction of MET × time; ^6^ Interaction of CHOL × time; ^7^ Branched-chain AA; ^8^ Non-essential AA; ^9^ Total AA.

**Table 2:** Plasma non-proteinogenic AA and AA derivatives concentrations during the periparturient period in cows supplemented with or without rumen-protected MET and CHOL.

	MET		CHOL			Day				*p*-Value ^2^				
Parameter (µM)	Without	With	Without	With	SEM ^1^	−10	4	14	28	MET ^3^	CHOL ^4^	Time	M × T ^5^	C × T ^6^
AA and derivatives ^#^														
1-methyl histidine	15.36	16.56	16.79	15.15	0.57	12.82 ^c^	12.58 ^c^	18.38 ^b^	21.85 ^a^	0.12	0.04	<0.01	0.36	0.50
3-methyl histidine	6.44	6.48	6.97	5.99	0.29	5.21 ^c^	11.00 ^a^	6.91 ^b^	4.40 ^d^	0.92	0.02	<0.01	0.12	0.75
α-aminoadipic acid	7.37	8.15	8.03	7.48	0.36	6.09 ^d^	6.99 ^c^	8.65 ^b^	9.81 ^a^	0.12	0.27	<0.01	0.91	0.30
α-aminobutyric acid	15.64	19.78	18.13	17.07	0.81	8.94 ^c^	23.60 ^a^	22.77 ^a^	19.93 ^b^	<0.01	0.32	<0.01	0.61	0.44
β-alanine	9.06	8.87	8.81	9.11	0.32	8.97	8.69	9.24	8.94	0.68	0.50	0.54	0.96	0.92
γ-aminobutyric acid	2.42	3.49	2.46	3.45	0.29	1.88 ^b^	3.08 ^a^	3.44 ^a^	3.41 ^a^	0.01	0.02	<0.01	0.27	0.75
Carnosine *	15.88	19.92	16.60	19.07	1.29	16.74 ^c^	14.20 ^d^	18.71 ^b^	22.51 ^a^	0.02	0.14	<0.01	0.30	0.02
Citrulline	71.19	83.47	79.08	75.14	2.92	69.61 ^c^	63.69 ^c^	82.86 ^b^	96.11 ^a^	<0.01	0.30	<0.01	0.99	0.60
Glutathione	4.68	4.73	4.84	4.57	0.27	4.60 ^b^	6.11 ^a^	4.35 ^bc^	3.76 ^c^	0.89	0.50	<0.01	0.50	0.74
Hydroxylysine	0.32	0.28	0.30	0.29	0.04	0.33	0.29	0.33	0.25	0.37	0.79	0.21	0.61	0.70
Hydroxyproline	15.19	15.36	15.42	15.13	0.48	12.80 ^c^	18.10 ^a^	16.31 ^b^	13.89 ^c^	0.81	0.67	<0.01	0.94	0.12
Ornithine	29.14	32.00	30.48	30.60	1.35	39.37 ^a^	22.11 ^d^	30.04 ^c^	33.25 ^b^	0.13	0.95	<0.01	0.12	0.45
Phosphoserine	6.22	6.18	6.07	6.33	0.18	5.53 ^c^	5.91 ^bc^	6.72 ^ab^	6.74 ^a^	0.88	0.30	<0.01	0.71	0.91
Sarcosine	10.65	11.43	11.50	10.58	0.56	8.32 ^b^	11.31 ^a^	12.03 ^a^	12.50 ^a^	0.33	0.27	<0.01	0.76	0.37
Urea	4121.63	4442.94	4323.55	4235.16	123.89	4104.53 ^bc^	4317.26 ^b^	4036.81 ^c^	4687.49 ^a^	0.07	0.60	<0.01	0.55	0.62
Sulfur-containing compounds														
Cystathionine	1.60	2.12	1.84	1.89	0.07	2.30	1.51	1.71	1.92	<0.01	0.61	<0.01	0.08	0.36
Cystine	7.56	9.58	7.65	9.48	34.5	11.20 ^a^	6.41 ^d^	7.73 ^c^	8.92 ^b^	<0.01	0.02	<0.01	0.01	0.17
Homocystine	4.17	5.10	4.52	4.70	0.30	4.80 ^a^	3.99 ^b^	4.80 ^a^	4.92 ^a^	0.02	0.65	0.01	0.69	0.28
Taurine	33.23	47.04	39.43	40.84	1.28	34.51	40.49	40.06	45.48	<0.01	0.44	<0.01	0.16	0.92
TSC ^7^	70.65	97.37	82.47	85.95	1.93	80.52	84.03	83.93	87.57	<0.01	0.44	0.08	0.07	0.48
Met%TSC	28.44	31.4	31.09	28.74	0.72	29.65 ^b^	32.35 ^ab^	31.15 ^b^	26.51 ^c^	0.01	0.03	<0.01	0.74	0.24

* Significant (*p* < 0.05) parity effect observed. Labeled means in a row without a common superscript letter differ (*p* < 0.05). ^#^ β-aminobutyric acid undetectable; ^1^ Greatest SEM; ^2^ MET × CHOL interaction was included in the supplemental file; ^3^ Overall effect of MET supplementation; ^4^ Overall effect of CHOL supplementation; ^5^ Interaction of MET × time; ^6^ Interaction of CHOL × time; ^7^ Total sulfur-containing AAs and derivatives (methionine, cystathionine, cystine, homocysteine, and taurine).

**Table 3:** Hepatic relative *PC*^#^ and *PCK1* mRNA expression during the periparturient period in cows supplemented with or without rumen-protected MET and CHOL.

	MET		CHOL			Day				*p*-Value ^2^				
Genes	Without	With	Without	With	SEM ^1^	−10	7	20	30	MET ^3^	CHOL ^4^	Time	M × T ^5^	C × T ^6^
*PC*	2.12	2.02	2.09	2.05	0.07	1.49 ^c^	2.88 ^a^	2.23 ^b^	1.93 ^d^	0.33	0.70	<0.01	0.28	0.34
*PCK1*	2.27	2.43	2.39	2.31	0.10	1.57 ^c^	2.62 ^ab^	2.74 ^a^	2.69 ^ab^	0.27	0.55	<0.01	0.48	0.46

* Significant (*p* < 0.05) parity effect observed. Labeled means in a row without a common superscript letter differ (*p* < 0.05). ^#^ *PC* = pyruvate carboxylase; *PCK1* = Phosphoenolpyruvate carboxykinase 1; ^1^ Greatest SEM; ^2^ MET × CHOL interaction was included in the supplemental file; ^3^ Overall effect of MET supplementation; ^4^ Overall effect of CHOL supplementation; ^5^ Interaction of MET × time; ^6^ Interaction of CHOL × time.

## Supplementary Materials

The following are available online at http://www.mdpi.com/2072-6643/9/1/10/s1, RNA Extraction and mRNA Expression Calculation, Table S1: Plasma proteinogenic AA concentrations during the transition period in cows supplemented with or without rumen-protected methionine (MET) and choline (CHOL), Table S2: Plasma non-proteinogenic AA and AA derivatives concentrations during the transition period in cows supplemented with or without rumen-protected MET and CHOL, Table S3: Hepatic relative *PC* and *PCK1* mRNA expression during the transition period in cows with or without rumen-protected MET and CHOL.
