# Supplementary material for: Methionine and Choline Supply during the Periparturient Period Alter Plasma Amino Acid and One-Carbon Metabolism Profiles to Various Extents: Potential Role in Hepatic Metabolism and Antioxidant Status
Source: Nutrients. 2016 Dec 29;9(1):10. doi: 10.3390/nu9010010 (PMC5295054; doi:10.3390/nu9010010)
Supplement: Supplementary file 1 [file nutrients-09-00010-s001.docx]

Supplementary Materials: Methionine and Choline Supply during the Periparturient Period Alter Plasma Amino Acid and One-Carbon Metabolism Profiles to Various Extents: Potential Role in Hepatic Metabolism and Antioxidant Status

Zheng Zhou, Mario Vailati-Riboni, Daniel N. Luchini and Juan J. Loor

RNA Extraction and mRNA Expression Calculation

***Liver RNA extraction.*** Liver tissue (30–50 mg) was homogenized in a TissueLyser II (Qiagen, Redwood, CA, USA) for 2 × 2 min bursts at 30 Htz. Total RNA and DNA were extracted using a Qiagen AllPrep DNA/RNA mini kit (Qiagen, Redwood, CA, USA) as per manufacturer’s instructions and RNA was treated with DNase (Ambion DNA-free kit; Ambion Inc., Austin, TX, USA).

***mRNA expression calculation.*** Efficiency of qPCR amplification for each gene was calculated using the standard curve method (Efficiency = 10^(–1/slope)^). The mRNA expression of the measured genes was calculated as previously reported [1], using the inverse of PCR efficiency raised to Δ*C*_t_ (gene abundance = 1/EΔ*C*_t_, where Δ*C*_t_ = *C*_t_ of tested gene—geometric mean *C*_t_ of 3 internal control genes). Overall mRNA expression for each gene among all samples of the same liver tissue was calculated using the median Δ*C*_t_, and overall percentage of each mRNA as a proportion of measured genes was computed from the equation: 100× mRNA abundance of each individual gene/sum of mRNA abundance of all the genes investigated.

***Quantitative RT-PCR (qPCR).*** The qPCR was performed in a MicroAmp Optical 384-Well Reaction Plate (Cat. #4309849, Applied Biosystems, Carlsbad, CA, USA). Within each well, 4 μL of diluted cDNA combined with 6 μL of mixture composed of 5 μL 1× SYBR Green master mix (Cat. #4309155, Applied Biosystems), 0.4 μL each of 10 μM forward and reverse primers, and 0.2 μL of DNase/RNase-free water were added. Three replicates and a 6-point standard curve plus the nontemplate control (NTC) were run for each sample to test the relative expression level. qPCR was conducted in ABI Prism 7900 HT SDS instrument (Applied Biosystems, Carlsbad, CA, USA) following the conditions below: 2 min at 50 °C, 10 min at 95 °C, 40 cycles of 15 s at 95 °C (denaturation), and 1 min at 60 °C (annealing + extension). The presence of a single PCR product was verified by the dissociation protocol using incremental temperatures to 95 °C for 15 s, then 65 °C for 15 s. The threshold cycle (*C*_t_) data were analyzed and transformed using the standard curve with the 7900 HT Sequence Detection System Software (version 2.2.1, Applied Biosystems, CA, USA). Data were then normalized with the geometric mean of the three Internal Control Genes (ICG).

**Table S1.** Plasma proteinogenic AA concentrations during the transition period in cows supplemented with or without rumen-protected methionine (MET) and choline (CHOL).

|  | **Treatments** | | | |  | ***p*-Value** | |
| --- | --- | --- | --- | --- | --- | --- | --- |
| **AA (µM)** | **CON ^1^** | **SMA** | **REA** | **MIX** | **SEM ^2^** | **M × C ^3^** | **M × C × T ^4^** |
| Essential AA |  |  |  |  |  |  |  |
| Arginine | 54.61 | 61.5 8 | 54.89 | 59.31 | 2.52 | 0.61 | 0.31 |
| Histidine | 52.94 | 55.17 | 51.83 | 53.94 | 1.69 | 0.98 | 0.17 |
| Isoleucine | 97.84 | 103.50 | 93.36 | 103.72 | 6.28 | 0.69 | 0.99 |
| Leucine | 151.85 | 153.56 | 154.77 | 170.47 | 7.55 | 0.46 | 0.69 |
| Lysine | 59.28 | 67.94 | 62.74 | 69.55 | 3.56 | 0.75 | 0.82 |
| Methionine | 18.88 | 29.21 | 18.42 | 28.68 | 1.19 | 0.97 | 0.46 |
| Phenylalanine | 47.41 ^b^ | 44.40 ^b^ | 45.87 ^b^ | 49.94 ^a^ | 1.50 | 0.02 | 0.56 |
| Threonine | 75.65 | 76.20 | 69.46 | 81.13 | 3.56 | 0.12 | 0.52 |
| Tryptophan | 21.56 | 23.80 | 22.95 | 26.28 | 0.91 | 0.55 | 0.49 |
| Valine | 225.27 | 233.12 | 228.27 | 256.52 | 13.80 | 0.45 | 0.95 |
| BCAA^5^ | 476.13 | 491.42 | 477.22 | 531.31 | 29.33 | 0.49 | 0.91 |
| EAA^6^ | 831.48 | 866.95 | 822.75 | 925.45 | 35.85 | 0.08 | 0.32 |
| Met%EAA | 2.26 | 3.30 | 2.26 | 3.07 | 0.14 | 0.40 | 0.63 |
| Non-essential AA |  |  |  |  |  |  |  |
| Alanine | 191.99 | 204.74 | 182.78 | 208.00 | 7.89 | 0.43 | 0.28 |
| Asparagine | 36.39 | 39.22 | 33.46 | 40.05 | 2.11 | 0.33 | 0.21 |
| Aspartate | 3.85 | 4.68 | 4.62 | 4.90 | 0.30 | 0.26 | 0.01 |
| Glutamate | 34.91 | 38.05 | 36.99 | 38.64 | 1.47 | 0.58 | 0.02 |
| Glutamine | 251.94 | 263.91 | 240.5 | 255.15 | 9.01 | 0.88 | 0.38 |
| Glycine | 434.39 | 420.4 | 419.27 | 392.03 | 21.53 | 0.76 | 0.95 |
| Proline | 72.63 | 75.44 | 71.97 | 81.04 | 2.88 | 0.29 | 0.26 |
| Serine | 90.31 | 87.02 | 82.94 | 90.81 | 3.23 | 0.09 | 0.20 |
| Tyrosine | 41.82 ^ab^ | 39.75 ^b^ | 39.82 ^b^ | 47.04 ^a^ | 2.17 | 0.04 | 0.10 |
| NEAA^7^ | 1134.23 | 1173.34 | 1095.29 | 1130.23 | 35.65 | 0.97 | 0.38 |
| TAA^8^ | 1952.08 | 2040.49 | 1902.13 | 2065.68 | 60.15 | 0.51 | 0.31 |
| Met%TAA | 0.94 | 1.39 | 0.94 | 1.34 | 0.06 | 0.67 | 0.69 |
| Lys%TAA | 3.08 | 3.45 | 3.35 | 3.49 | 0.15 | 0.43 | 0.99 |

*** Significant (*p* < 0.05) parity effect observed; ^1^ CON = control; SMA = Smartamine M (0.08% of DM); REA = ReaShure (60 g/days); MIX = SMA + REA; ^2^ Greatest SEM; ^3^ Interaction of MET × CHOL;
^4^ Interaction of MET × CHOL × Time; ^5^ Branched-chain AA; ^6^ Essential AA; ^7^ Non-essential AA; ^8^ Total AA.

**Table S2.** Plasma non-proteinogenic AA and AA derivatives concentrations during the transition period in cows supplemented with or without rumen-protected MET and CHOL.

|  | **Treatments** | | | |  | ***p*-Value** | | |
| --- | --- | --- | --- | --- | --- | --- | --- | --- |
| **Parameter (µM)** | **CON ^1^** | **SMA** | **REA** | **MIX** | **SEM ^2^** | **M × C ^3^** | **M × C × T ^4^** | |
| AA and derivatives ^#^ |  |  |  |  |  |  |  | |
| 1-methyl histidine | 16.30 | 17.29 | 14.48 | 15.86 | 0.82 | 0.73 | 0.50 | |
| 3-methyl histidine | 6.85 | 7.09 | 6.06 | 5.92 | 0.42 | 0.63 | 0.90 | |
| α-aminoadipic acid | 7.57 | 8.52 | 7.18 | 7.80 | 0.53 | 0.78 | 0.90 | |
| α-aminobutyric acid | 15.99 | 20.56 | 15.31 | 19.04 | 1.22 | 0.78 | 0.61 | |
| β-alanine | 9.08 | 8.54 | 9.04 | 9.19 | 0.45 | 0.45 | 0.71 | |
| γ-aminobutyric acid | 1.86 | 3.07 | 2.99 | 3.91 | 0.42 | 0.73 | 0.59 | |
| Carnosine * | 14.62 | 18.84 | 17.25 | 21.08 | 1.27 | 0.78 | 0.64 | |
| Citrulline | 73.50 | 85.08 | 68.96 | 81.88 | 4.13 | 0.79 | 0.71 | |
| Glutathione | 4.48 | 5.20 | 4.88 | 4.26 | 0.40 | 0.09 | 0.43 | |
| Hydroxylysine | 0.34 | 0.27 | 0.30 | 0.28 | 0.06 | 0.62 | 0.90 | |
| Hydroxyproline | 15.35 | 15.50 | 15.04 | 15.22 | 0.71 | 0.98 | 0.76 | |
| Ornithine | 29.60 | 31.38 | 28.69 | 32.64 | 1.95 | 0.56 | 0.63 | |
| Phosphoserine | 5.91 | 6.23 | 6.55 | 6.13 | 0.27 | 0.15 | 0.19 | |
| Sarcosine | 11.35 | 11.65 | 9.95 | 11.20 | 0.83 | 0.54 | 0.88 | |
| Urea | 4169.91 | 4483.16 | 4073.91 | 4402.78 | 176.54 | 0.95 | 0.73 | |
| Sulfur-containing compounds | | | | | | | |  |
| Cystathionine | 1.55 | 2.12 | 1.65 | 2.12 | 0.10 | 0.57 | 0.64 | |
| Cystine * | 6.56 | 8.75 | 8.54 | 10.41 | 0.43 | 0.70 | 0.97 | |
| Homocystine | 4.22 | 4.85 | 4.13 | 5.36 | 0.45 | 0.49 | 0.26 | |
| Taurine | 30.57 ^c^ | 48.29 ^a^ | 35.89 ^b^ | 45.80 ^a^ | 1.81 | 0.04 | 0.53 | |
| TSC ^5^ | 66.74 ^c^ | 99.16 ^a^ | 74.57 ^b^ | 95.58 ^a^ | 2.73 | 0.04 | 0.13 | |
| Met%TSC | 30.27 | 31.92 | 26.61 | 30.88 | 1.06 | 0.21 | 0.94 | |

*** Significant (*p* < 0.05) parity effect observed; ^#^ β-aminobutyric acid undetectable; ^1^ CON = control; SMA = Smartamine M (0.08% of DM); REA = ReaShure (60 g/days); MIX = SMA + REA; ^2^ Greatest SEM; ^3^ Interaction of MET × CHOL; ^4^ Interaction of MET × CHOL × Time; ^5^ Total sulfur-containing AAs and derivatives (methionine, cystathionine, cystine, homocysteine, and taurine).

**Table S3.** Hepatic relative *PC*^#^ and *PCK1* mRNA expression during the transition period in cows with or without rumen-protected MET and CHOL.

|  | **Treatments** | | | |  | ***p*-Value** | |
| --- | --- | --- | --- | --- | --- | --- | --- |
| **Gene** | **CON ^1^** | **SMA** | **REA** | **MIX** | **SEM ^2^** | **M × C ^3^** | **M × C × T ^4^** |
| *PC* | 2.13 | 2.05 | 2.11 | 1.99 | 0.11 | 0.87 | 0.77 |
| *PCK1* | 2.39 | 2.39 | 2.16 | 2.46 | 0.14 | 0.26 | 0.10 |

*** Significant (*p* < 0.05) parity effect observed; ^#^ *PC* = *pyruvate carboxylase*; *PCK1* = *Phosphoenolpyruvate carboxykinase 1*; ^1^ CON = control; SMA = Smartamine M (0.08% of DM); REA = ReaShure (60 g/days); MIX = SMA + REA; ^2^ Greatest SEM; ^3^ Interaction of MET × CHOL; ^4^ Interaction of MET × CHOL × Time; ^5^ Total sulfur-containing AAs and derivatives (methionine, cystathionine, cystine, homocysteine, and taurine).

References

1. Shahsavari, A.; D’Occhio, M.J.; Al Jassim, R. The role of rumen-protected choline in hepatic function and performance of transition dairy cows. *Br. J. Nutr.* **2016**, *116*, 35–44.

© 2016 by the authors; licensee MDPI, Basel, Switzerland. This article is an open access article distributed under the terms and conditions of the Creative Commons by Attribution (CC-BY) license (http://creativecommons.org/licenses/by/4.0/).
